# Does the accelerometer-based navigation system reduce blood loss and transfusion in one-stage sequential bilateral total knee arthroplasty? A randomized double-blind controlled trial

**DOI:** 10.1186/s12891-023-06648-8

**Published:** 2023-06-29

**Authors:** Atthakorn Jarusriwanna, Chaturong Pornrattanamaneewong, Rapeepat Narkbunnam, Pakpoom Ruangsomboon, Paweena Thitithapana, Keerati Chareancholvanich

**Affiliations:** 1 Department of Orthopaedics, Faculty of Medicine, Naresuan University, Phitsanulok, Thailand; 2 Department of Orthopaedic Surgery, Faculty of Medicine Siriraj Hospital, Mahidol University, Bangkok, Thailand; 3 Department of Anesthesiology, Phyathai 3 Hospital, Bangkok, Thailand

**Keywords:** Sequential bilateral total knee arthroplasty, Accelerometer-based navigation, Complications, Blood loss, Blood transfusion

## Abstract

**Background:**

Total knee arthroplasty (TKA) is associated with significant blood loss and postoperative transfusion. The accelerometer-based navigation (ABN) system guides the bone cutting plane without breaching the intramedullary canal, which may reduce bleeding. This study aimed to investigate blood loss and transfusion compared between the ABN system and the conventional procedure in patients undergoing one-stage sequential bilateral TKA (SBTKA).

**Methods:**

A total of 66 patients scheduled for SBTKA were randomly allocated to either the ABN or conventional group. Postoperative hematocrit (Hct) level, drainage blood loss, transfusion rate, and amount of packed red cell transfusion were collected. Total red blood cell (RBC) loss was then calculated for the primary outcome.

**Results:**

The mean calculated total RBC loss in the ABN and conventional group was 669.7 and 630.0 mL, respectively (*p* = 0.572). There was no significant difference between groups for other evaluated outcome parameters, including postoperative Hct level, drainage blood loss, or packed red cell transfusion volume. All patients in the conventional group required postoperative blood transfusion while 96.8% of patients in the ABN group were transfused.

**Conclusions:**

The total RBC loss and volume of packed red cells transfusion were not significant difference between interventions, which suggest no benefit of the ABN system in reducing blood loss and transfusion in patients undergoing SBTKA.

**Trial registration:**

The protocol of this study was registered in the Thai Clinical Trials Registry database no. TCTR20201126002 on 26/11/2020.

## Introduction

Blood loss is a complication of considerable concern following total knee arthroplasty (TKA), especially in the early postoperative period. This condition can lead to hemodynamic instability and requires blood transfusion in those who become hypovolemic [1]. Previous studies reported allogenic blood transfusion to be associated with an increased rate of infections, volume overload, immunologic reactions, and overall mortality [2, 3], and these types of problems adversely affected postoperative ambulation and delayed functional rehabilitation after TKA [4]. The reported volume of blood loss and the proportion of patients requiring transfusion among patients who underwent unilateral TKA varied from 500 to 2,000 mL for total blood loss [5–8], and from 3 to 20% of patients requiring blood transfusion [9, 10]. Patients who underwent one-stage sequential bilateral TKA (SBTKA) were reported to have greater blood loss and transfusion with an approximate 5.51 times higher bleeding risk [11], and an almost two times higher blood transfusion rate [12].

There are two forms of blood loss in TKA. The first is visible blood loss, which is defined as blood loss in the surgical field and drainage. The second is invisible or hidden blood loss, which refers to blood loss within tissues, the intramedullary canal, and bony surfaces [6]. Previous studies reported hidden blood loss to comprise approximately 38–50% of total blood loss [6, 7]. The distal femoral cut in conventional TKA is usually performed using an intramedullary guide rod to reference the anatomical axis. This technique requires femoral canal breaching, which is suspected of being one of the main causes of perioperative blood loss [13]. The accelerometer-based navigation (ABN) device, which is a handheld computer-assisted accelerometer-based stereotaxic system, displays alignment information and guides the bone resection in the operative field. This system is characterized by an accelerometer gyroscopic wireless pod that is attached to the femoral resection jigs followed by calibration via a prescribed set of steps [14, 15]. After alignment verification, the femoral bone cut can be performed without violating the femoral canal [16]. Non-breaching of the femoral canal via the use of the ABN system may reduce hidden blood loss during and after TKA.

Data specific to blood loss and transfusion rate between the conventional technique and the non-breaching intramedullary canal technique in patients undergoing one-stage SBTKA remain scarce. Accordingly, the aim of this study was to investigate blood loss and transfusion compared between the ABN system and the conventional procedure in patients undergoing one-stage SBTKA.

## Materials and methods

The protocol of this randomized double-blind controlled trial was registered in the Thai Clinical Trials Registry (registration no. TCTR20201126002). Written informed consent was obtained from all study participants. Patients diagnosed with bilateral knee osteoarthritis who were scheduled for primary one-stage SBTKA between 2020 and 2021 were included in this study. Patients having one or more of the following were excluded: history of any previous knee surgery, knee infection, bleeding disorder or thromboembolic event, a diagnosis of secondary osteoarthritis, and/or hypersensitivity to any medication in the study protocol (bupivacaine, tranexamic acid, parecoxib, and morphine sulfate).

### Surgical procedure and outcome measurement

**Fig. 1:**
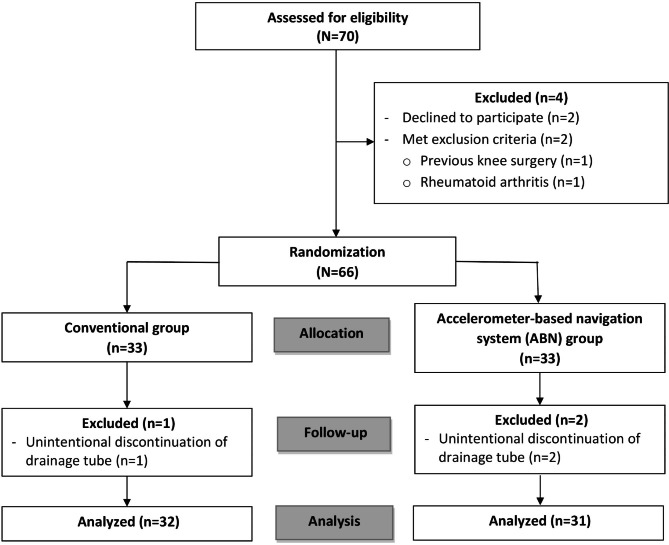
Consolidated Standards of Reporting Trials (CONSORT) diagram showing the enrollment and flow of patients in this study

Seventy patients who were scheduled for primary SBTKA were assessed for eligibility. Of those, four patients were excluded (two patients declined to participate in the study, one patient had previous knee surgery, and another patient had underlying rheumatoid arthritis). The remaining 66 patients were formally enrolled in the study (Fig. 1). All surgical procedures were performed by a single surgeon under spinal anesthesia with bupivacaine. The enrolled patients were randomized using a computer-generated sequential block-of-four randomization technique. Each of the 66 group allocations were kept in sequential sealed envelopes that were opened before surgery to determine whether the patient would undergo ABN or the conventional SBTKA technique.

In the conventional group, the distal femoral bone cut was made after insertion of the intramedullary reference guide into the intercondylar notch, after which the opening of the intramedullary canal was occluded with a bone plug before prosthesis implantation [17]. The ABN group underwent the distal femoral bone cut guided by an ABN device (iASSIST®; Zimmer CAS, Montreal, Canada) without breaching the intramedullary canal. The proximal tibial bone cut was performed using the extramedullary reference guide in both groups. The first of the two knees to be operated upon was determined by patient preference. A uniform tourniquet inflation pressure of 300 mmHg was inflated at the thigh of the operated knee before skin incision and deflated after skin closure. Ten mg/kg intravenous tranexamic acid was given to all patients during the time between tourniquet inflation and skin incision of the first knee and again 3 h after both knees had been operated upon [18, 19]. A cemented, fixed-bearing, posterior-stabilized knee prosthesis (NexGen LPS; Zimmer Biomet, Warsaw, IN, USA) was implanted via a standard medial parapatellar approach in all knees.

After surgery, a non-compressive dressing was applied to all knees [20]. The drainage tubes were managed using the 3-hour interval clamping technique [21]. During the first 3 h after surgery, the drainage tubes were clamped to create a tamponade effect, followed by clamp release for the next 3 h, and followed by reclamping for the next 3 h. After this interval, the drainage tubes were left continuously unclamped and then removed at 48 h after surgery. Postoperative physical therapy and rehabilitation, including the use of a continuous passive motion (CPM) device and early ambulation with a gait aid, was encouraged. An intermittent pneumatic compression device was applied to all patients during hospital stay for mechanical venous thromboembolism (VTE) prophylaxis. No anticoagulants were given for routine medical VTE prophylaxis since the benefit of this anti-VTE strategy has not yet to be established in Asian populations [22]. Intravenous 40 mg of parecoxib was given for pain every 12 h until the 48-hour postoperative time point, and intravenous 3 mg of morphine sulfate was injected as needed for breakthrough pain every 4 h if the 10 cm visual analog scale (VAS) score was above 5. Complete blood count (CBC), including hemoglobin (Hb) and hematocrit (Hct), was collected prior to surgery and at 24, 48, and 120 h postoperatively. Drainage blood loss was recorded at 24 and 48 h after surgery. Packed red cells were transfused if the patient had one of these conditions: Hb level < 9 g/dL; clinical symptoms of acute anemia, including syncope, dizziness, mucocutaneous pallor, unstable vital signs, and/or urine output < 0.5 mL/kg/hr [23]. The primary outcome in this study was total red blood cell (RBC) loss as calculated using the Merculiari formula, which is based on the preoperative Hct and Hct at postoperative day 5, as well as the volume of transfused packed RBCs and the patient’s blood volume before surgery [24]. The Merculiari formula is described as follows: total RBC loss (mL) = total blood volume (mL) x (Hct_pre−op_ – Hct_post−op day 5_) + transfused RBC (mL). Total blood volume was calculated using the Nadler formula, which takes into account gender, weight and height, as follows: total blood volume (mL) = 1,000 x [(k1 x height (m)^3^) + (k2 x weight (kg)) + k3] (when k1 = 0.3669, k2 = 0.03219, and k3 = 0.6041 for men; and k1 = 0.3561, k2 = 0.03308, and k3 = 0.18331 for women, respectively) [25].

The intensity of postoperative pain at rest after physical therapy was assessed by VAS pain score (range: 0–10). Total morphine use and length of hospital stay were also collected. All postoperative outcomes were recorded by a group of assessors who were blinded to the randomization and the study intervention.

### Sample size calculation and statistical analysis

The sample size for this study was calculated using the mean and standard deviation data from a previously published study that investigated bleeding in patients who underwent SBTKA compared between robotic-assisted technique on one side and conventional technique on the contralateral side [26]. Our calculation revealed that a sample size of 30 patients in each group would yield 80% statistical power to detect a significance level of 0.05. To compensate for a possible 10% dropout rate for any reason, the target enrollment number for each group was 33 patients.

All statistical analyses were performed using PASW Statistics software (SPSS Inc., Chicago, IL, USA). The normality of continuous data was evaluated using Kolmogorov-Smirnov test. Normally distributed continuous variables were compared using Student’s *t*-test, and categorical factors were compared using chi-square test or Fisher’s exact test depending on the size of the sample. Wilcoxon matched-pairs signed rank test was used to compare non-normally distributed continuous data. All factors compared between groups are shown as mean plus/minus standard deviation for normally distributed continuous variables, and as frequency and percentage for categorical variables. Statistical significance was defined as a *p*-value less than 0.05.

## Results

Among the 66 patients who were enrolled in this study, 3 patients were excluded from the final analysis due to unintentional discontinuation of the drainage tube before the 48-hour time point after surgery. The remaining 63 patients (32 patients in the conventional group and 31 patients in the ABN group) completed the study (Fig. 1). The mean age of overall participants in the study was 70.5 years, and 90.5% were female. The baseline characteristics, including age, gender, body mass index (BMI), comorbidities as assessed by the Charlson Comorbidity Index (CCI), preoperative physical function as evaluated by the Knee Injury and Osteoarthritis Outcome Score (KOOS), total blood volume, preoperative Hct level, platelet count, and total intraoperative tourniquet time, were not significantly different between groups (Table 1).

**Table 1:** Preoperative demographics and perioperative characteristics of study patients compared between the conventional group and the ABN group

Characteristics	Conventional group (n = 32) Mean ± SD or n (%)	ABN group (n = 31) Mean ± SD or n (%)	*p*-value
Age (years)	70.0 ± 6.6	70.9 ± 6.0	0.568
Gender			0.634
Female	30 (90.9%)	27 (87.1%)	
Male	3 (9.1%)	4 (12.9%)	
Body mass index (kg/m^2^)	28.1 ± 4.1	26.7 ± 3.3	0.152
Charlson Comorbidity Index			0.519
0 to 1	24 (72.7%)	21 (67.7%)	
≥2	9 (27.3%)	10 (32.3%)	
Preoperative KOOS	38.5 ± 14.1	37.9 ± 12.4	0.865
Total blood volume (mL)	2,912.5 ± 412.2	2,906.5 ± 383.9	0.952
Preoperative hematocrit level (%)	40.6 ± 3.1	38.6 ± 4.1	0.053
Preoperative platelet count (x10^3^/mm^3^)	245.0 ± 75.7	244.2 ± 51.7	0.960
Total intraoperative tourniquet time (minutes)	139.3 ± 26.5	151.7 ± 26.0	0.070

A *p*-value < 0.05 indicates statistical significance.

Abbreviations: ABN, accelerometer-based navigation system; KOOS, Knee Injury and Osteoarthritis Outcome Score; SD, standard deviation

Regarding postoperative outcomes, there was no significant difference between groups relative to postoperative Hct level at 24, 48, and 120 h after surgery. Drainage blood loss at 24 and 48 h, and total drainage blood loss were all lower in the ABN group than in the conventional group, but all 3 of those differences failed to achieve statistical significance. Concerning the primary outcome, the mean calculated total RBC loss was 630.0 ± 277.8 mL (range: 266.0–1,282.1) and 669.7 ± 279.7 mL (range: 302.3–1,290.7) in the conventional group and ABN group, respectively (*p =* 0.572). All patients in the conventional group required packed red cell transfusion, whereas 96.8% of patients in the ABN group were transfused (*p* = 0.492). The packed red cell transfusion volume at any evaluated time point and the total volume were not significantly different between groups (Table 2).

**Table 2:** Postoperative outcome variables compared between the conventional group and the ABN group

Clinical variables	Conventional group (n = 32) Mean ± SD or n (%)	ABN group (n = 31) Mean ± SD or n (%)	*p*-value
Postoperative hematocrit level (%)			
24 h	29.9 ± 2.8	29.6 ± 3.2	0.723
48 h	30.2 ± 2.4	30.1 ± 3.1	0.906
120 h	32.8 ± 2.1	33.1 ± 1.9	0.537
Drainage blood loss (mL)			
24 h	536.8 ± 237.0	520.3 ± 228.6	0.778
48 h	397.3 ± 364.2	313.9 ± 204.4	0.260
Total	934.1 ± 535.5	834.2 ± 304.1	0.359
Calculated total red blood cell loss (mL)	630.0 ± 277.8	669.7 ± 279.7	0.572
Patients that required blood transfusion	32 (100%)	30 (96.8%)	0.492
Packed red cell transfusion volume (mL)			
24 h	247.2 ± 163.7	265.2 ± 124.6	0.626
48 h	241.4 ± 167.0	211.4 ± 148.6	0.451
After 48 h	118.3 ± 147.9	176.9 ± 170.8	0.148
Total	606.9 ± 277.9	653.5 ± 286.8	0.512
Total morphine use (mL)	8.5 ± 5.9	10.0 ± 6.0	0.292
Length of hospital stay (days)	5.9 ± 1.7	6.1 ± 2.0	0.644

A *p*-value < 0.05 indicates statistical significance.

Abbreviations: ABN, accelerometer-based navigation system; SD, standard deviation

**Fig. 2:**
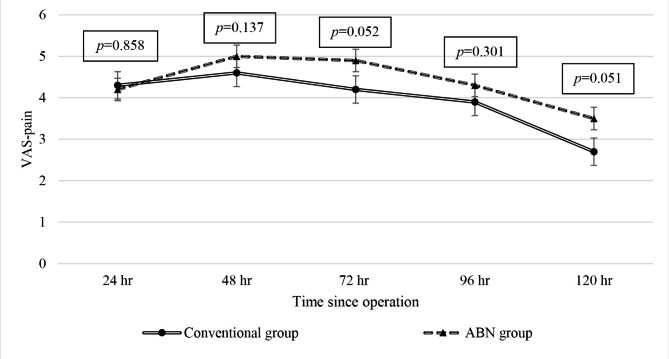
The mean postoperative VAS score compared between the conventional group and the ABN group at 24, 48, 72, 96, and 120 h after surgery. The difference in pain score was very close to being significantly higher in the ABN group at the 72-hour and 120-hour time points. A *p*-value ≤ 0.05 indicates statistical significance

Postoperative pain intensity tended to be lower in the conventional group with very close to statistically significant difference between groups observed at 72 and 120 h after surgery (higher pain score in the ABN group for both) (Fig. 2). There was no significant difference in total morphine use or length of hospital stay between groups (Table 2). No complications related to surgery, such as surgical site infection, clinical VTE, cardiovascular or cerebrovascular abnormalities, were observed in either group.

## Discussion

TKA is a surgical procedure that is associated with large volume of blood loss and increased postoperative blood transfusion. Various techniques to reduce perioperative visible and hidden blood loss, including intravenous injection of tranexamic acid with/without intra-articular combination, or applying topical hemostatic agent e.g., Floseal® at the surgical site, were shown to reduce total blood loss and blood transfusion in patients undergoing TKA or SBTKA [27–30]. However, the volume of blood loss remains high and blood transfusion is necessary in patients with hypovolemic complication. The aim of TKA with the ABN device and/or robotic-assisted surgery is to improve alignment accuracy and reliability of the prosthesis components [15]; furthermore, the advantage of being able to avoid violation of the intramedullary canal may confer a secondary advantage of reducing perioperative blood loss [13]. Prior study in unilateral TKA that compared blood loss between intramedullary non-violating device and the conventional intramedullary-violating technique demonstrated an inconclusive result. Kalairajah et al. reported a significant reduction in mean drainage blood loss of 396 mL and a mean calculated hemoglobin loss of 17 g/dL in patients undergoing computer-assisted TKA when compared to conventional TKA [31]. A randomized controlled study by Conteduca et al. found that patients who underwent computer-assisted TKA had an average estimated intraoperative blood loss of 297 mL lower than patients who underwent conventional TKA [32]. However, a recent systematic review and meta-analysis found no significant difference in postoperative blood loss-related indicators between ABN and conventional TKA [14].

Accordingly, this study is the first randomized double-blind controlled trial to investigate blood loss and transfusion compared between patients undergoing SBTKA with the ABN device and patients undergoing the conventional SBTKA technique. We used the Merculiari formula to calculate the estimated total RBC loss because of well reproducible and using the parameter which covers the overall perioperative period, including total blood volume and the blood transfusion volume. One of the strengths of this formula is comparison of blood-related parameters at specified time points (e.g., preoperative Hct compared to Hct at postoperative day 5) [24, 33, 34]. The results of our study revealed no significant difference in postoperative Hct level, drainage blood loss at any time point, the calculated total RBC loss, the need for blood transfusion, or the transfusion volume between SBTKA with ABN and conventional SBTKA. These findings seem to indicate that occlusion of the femoral canal opening with a bone plug in conventional TKA is as effective as the ABN system, which does not breach the intramedullary canal [17]. Nevertheless, the rate of blood transfusion was high in both groups as evidenced by the fact that only one patient in the entire study did not require postoperative blood transfusion.

A retrospective case-control study by Jhurani et al. found that patients who underwent SBTKA with computer navigation experienced no blood loss or blood transfusion benefit compared to those who underwent conventional SBTKA [35]. Similarly, a retrospective study by Laoruengthana et al. reported that ABN could not reduce blood loss or postoperative pain compared to conventional SBTKA [36]. A prospective randomized study by Song et al. reported that robotic-assisted TKA had significantly less postoperative mean drainage blood loss compared to conventional TKA among patients undergoing SBTKA [26]. However, every patients in their study underwent robotic-assisted TKA on one side and conventional TKA technique on the other side.

Intramedullary breaching and guide rod insertion in conventional TKA may stimulate pain and aggravate the inflammatory process in the medullary cavity [37, 38]. However, our study showed that patients in the conventional group had lower pain scores than patients in the ABN group in the early postoperative period, so the trend of longer operative time in the ABN group may associate with increased pain intensity [39]. Regarding the clinically significant difference in VAS pain score, a study by Kelly reported the minimum clinically significant difference (MCSD) in the VAS pain score to be 1.2 [40]. The maximum difference in the mean VAS pain score between groups in our study was 0.9 at 120 h after surgery.

This study has some mentionable limitations. First, the vast majority of subjects in this study were female, which may be a factor influencing blood loss during TKA. Prasad et al. reported that gender plays a role in postoperative blood loss, but not in intraoperative blood loss, and they found no significant difference in the amount of blood transfusion between genders [7]. Second, 3 enrolled patients (4.5%) were not included in the final analysis due to unintentional discontinuation of the drainage tube before the 48-hour postoperative time point. However, the remaining number of participants included in the final analysis would achieve the statistical power. Third and last, our decision to include intravenous tranexamic acid in the study protocol, and to exclude anticoagulants for medical VTE prophylaxis after surgery could have reduced the probability of perioperative bleeding when compared to the study protocols used in other published reports.

## Conclusions

The results of this study revealed no significant difference of total RBC loss and volume of packed red cells transfusion between ABN and conventional SBTKA, which suggests no benefit of the ABN system in reducing blood loss and transfusion. The conventional technique with bone plug occlusion at the femoral canal opening before prosthesis implantation is adequate for controlling bleeding during the procedure. Further study on the different protocol of perioperative bleeding control or the other non-breaching intramedullary canal technique e.g., robotic-assisted SBTKA may be warranted.

## Data Availability

The datasets used and/or analysed during the current study available from the corresponding author on reasonable request.
